# Rush Medical College

**Published:** 1866-12

**Authors:** 


### RUSH MEDICAL COLLEGE.

The commencement exercises of the *twenty-fourth* session of this College took place on the evening of October 3d.

The prospects of the school were never more flattering. A large class had already assembled, with every assurance that its members would be much augmented in a few days.

The whole appearance of the class, the expressions of earnestness, and evident manifestation of a desire on the part of its members to seek the knowledge which would best fit them for their duties as true physicians, could not but impress their teachers with the conviction that their labors would not be in vain.

No one could dream of the deep affliction which was so soon to fall upon those who were then assembled. In less than one week, the hand of death had taken away its most impressive lecturer and teacher, whose great intellect, deep wisdom, and almost unbounded professional experience, made him a tower of strength in the Institution he had founded so many years before.

In consequence of the death of Prof. Brainard, the exercises of the College were suspended for a few days.

At the present time, as we are informed by the Secretary of the Faculty, there is a larger class now present than ever before at a corresponding period of the course.

Friends of the school may be interested to know that the lectures on surgery are delivered by Prof. Powell.

In addition to the ordinary course of instruction as presented in the annual announcement, and the clinics described on another page, may be mentioned the "Quiz" class of Prof. Rae, and that of Drs. Powell, Lyman and Lackey.

